# Stimulation efficiency of an actuator driven piston at the biological interface to the inner ear

**DOI:** 10.1038/s41598-021-03195-x

**Published:** 2021-12-09

**Authors:** Susan Busch, Mohammad Ghoncheh, Thomas Lenarz, Hannes Maier

**Affiliations:** 1 Cluster of Excellence Hearing4all, Hannover, Germany; 2 Department of Otolaryngology, Hannover Medical School, Carl-Neuberg-Str. 1a, 30625 Hannover, Germany

**Keywords:** Biological physics, Outcomes research

## Abstract

Direct acoustic cochlear stimulation uses piston motion to substitute for stapes footplate (SFP) motion. The ratio of piston to stapes footplate motion amplitude, to generate the same loudness percept, is an indicator of stimulation efficiency. We determined the relationship between piston displacement to perceived loudness, the achieved maximum power output and investigated stapes fixation and obliteration as confounding factors. The electro-mechanical transfer function of the actuator was determined preoperatively on the bench and intraoperatively by laser Doppler vibrometry. Clinically, perceived loudness as a function of actuator input voltage was calculated from bone conduction thresholds and direct thresholds via the implant. The displacement of a 0.4 mm diameter piston required for a perception equivalent to 94 dB SPL at the tympanic membrane compared to normal SFP piston displacement was 27.6–35.9 dB larger, consistent with the hypothesis that the ratio between areas is responsible for stimulation efficiency. Actuator output was 110 ± 10 eq dB SPL_FF_ @1V_rms_ ≤ 3 kHz and decreased to 100 eq dB SPL_FF_ at 10 kHz. Output was significantly higher for mobile SFPs but independent from obliteration. Our findings from clinical data strongly support the assumption of a geometrical dependency on piston diameter at the biological interface to the cochlea.

## Introduction

Hearing aids (HAs) pick up sounds from the environment and present an amplified version of the sound to the ear canal. The acoustic properties of the HA loudspeaker are specified by the manufacturer and relevant individual properties, e.g. the Real-Ear-to-Coupler-Difference (RETCD) can be determined experimentally. In contrast to the well-defined acoustic output level in hearing aids, the evaluation of Active Middle Ear Implants (AMEI) is more complex. Although the signal processing and amplification are similar to hearing aids, the conversion of the output voltage to sound at the inner ear (cochlea) cannot be easily determined. For AMEIs that stimulate the ossicular chain, the ASTM standard^1^ provides an established method to determine the output in a temporal bone model. It was recently shown that results in the temporal bone model correspond well with clinical outcomes in patients^2^. However, this method cannot be used in direct acoustic stimulation of the cochlea, because the middle ear—required for the use of the ASTM standard—is circumvented, and the cochlea fluids are stimulated directly by an actuator.

**Figure 1:**
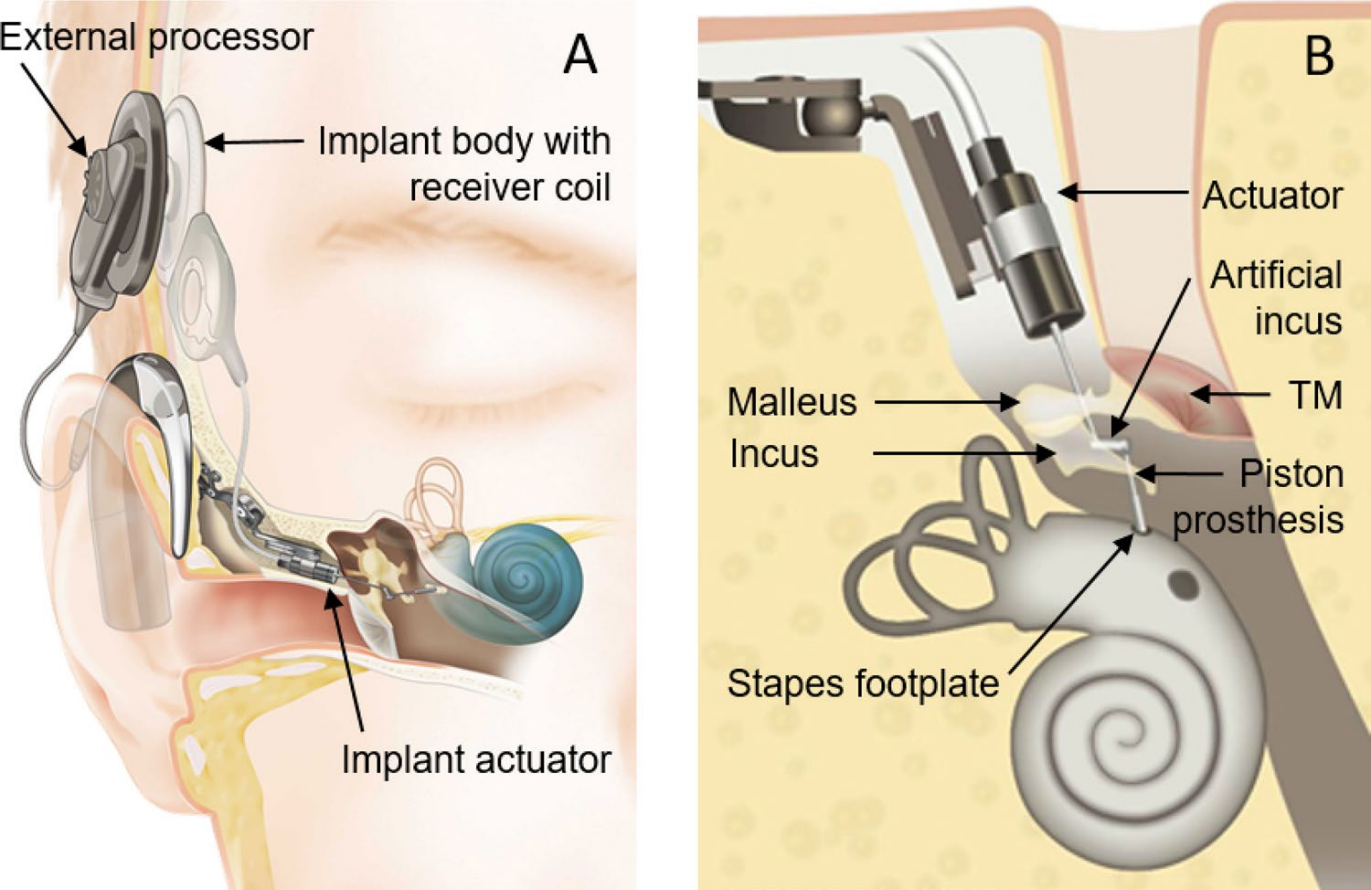
(**A**) Schematic of the Codacs direct acoustic cochlear stimulator consisting of an implant body with receiver coil, an actuator and an external processor stimulating the inner ear (**B**) actuator with artificial incus driving a piston prosthesis through a stapedotomy (image courtesy of Cochlear ltd.). *TM* tympanic membrane.

One device used for direct acoustic stimulation was the Cochlear Codacs Direct Acoustic Cochlear Implant System. It is a semi-implantable device consisting of an external sound processor and an implant that receives and decodes the sound information, and drives an implanted actuator that stimulates the cochlea directly (Fig. 1A). For this purpose, a conventional piston prosthesis, attached to the actuator, was used to stimulate the cochlear perilymph through a stapedotomy of the stapes footplate (Fig. 1B).

The piston has a much smaller area compared to the stapes footplate. Although the device was CE certified in 2013 (the CE mark is an acronym for “Conformité Européenne” that certifies that a product has met EU health and safety requirements) and thus approved for routine clinical use in the EU. However, the ratio of piston motion to equivalent stapes footplate motion that generates the same loudness percept was not determined up to now. The lower limit of the maximum power output (MPO) was estimated as > 112 dB HL in a small number of patients with a specialized experimental method^3,4^. However, quantifying the MPO with this method was not possible because it requires levels above the uncomfortable level (UCL) of the patients that may damage the inner ear. To our knowledge only one study with a similar experimental device investigated the output of piston stimulation by comparing round window volume displacement experimentally but did not explore the piston efficiency at the cochlear interface^5^. The ratio of the stimulation efficiency between naturally conducted sound via the stapes footplate and an actuator driven piston is crucial for the specification of future direct stimulation devices. In this study, we investigated the stimulation efficiency of an actuator driven piston in comparison to normal physiological sound transmission. For this purpose, we developed a new approach to determine the transfer function from piston (0.4 mm Fisch Teflon) displacement to perceived loudness from clinical patient data and investigated possible factors contributing to stimulation output levels.

## Methods

### Patients and demographics

At the Hannover Medical School (MHH), 77 ears of 74 subjects (three were bilaterally implanted) were implanted with a Cochlear Direct acoustic cochlear stimulator (Codacs) between Nov. 2009 and Nov. 2017. The mean age (± standard deviation) of the 46 females and 28 males at implantation was 62.5 ± 13.4 years. Of all implanted ears (44 right side and 33 left side), 13 ears underwent a subtotal petrosectomy with fatty tissue obliteration of the middle ear cavity at least 6 months before implantation. The stapes footplate (SFP) condition was classified as mobile or fixed by the surgeon during the implantation. A mobile SFP was found in 30 ears of which 9 ears were obliterated and 21 ears were not obliterated. A fixed SFP was found in 47 ears of which 4 ears underwent the obliteration procedure before implantation. An overview is given in Table 1.

**Table 1:** Distribution of the condition of the middle ear and stapes footplate (SFP) of the 77 ears (74 patients) that contributed data to the analysis.

	Mobile SFP	Fixed SFP
Obliterated	9	4
Not-obliterated	21	43

From 74 implanted subjects, intraoperative measurements of the actuator performance of 64 patients (27 males/37 females) with 66 implants (with two bilateral patients) were available and analyzed to determine the impact of implantation on actuator performance. The mean age in that group at implantation was 64.0 ± 13.6 years (N = 66 ears). In our cohort, 34 right ears and 32 left ears were implanted.

All patient data was acquired during routine measurements and the analysis was performed in retrospective from patient files and intraoperatively recorded data. Informed consent for anonymous use of data was obtained at the admission of patients. The data processing was done anonymous in accordance to the Regulation (EU) 2016/679 of 27 April 2016 on the protection of natural persons with regard to the processing of personal data, relevant guidelines and regulations and the internal regulations of the institution (MHH). According to German Data Protection and Professional Laws an ethics committee approval was not necessary for this study design as confirmed in a written statement by the local ethics committee.

### Data analysis

The goal of our analysis was to determine the efficiency of a direct piston stimulation of the cochlea, i.e. the mechano-acoustical transfer function

1 $${H}_{M-SPL}={p}_{T} [SPL]/d[\mathrm{\mu m}]$$

that describes the frequency specific relationship of a vibration displacement (d) of the piston to a percept, equivalent to an acoustic sound pressure level *p*_*T*_ at the tympanic membrane. In all patients in Hannover the Codacs was implanted in conjunction with a 0.4 mm diameter Fisch Teflon prosthesis, hence our analysis here is limited to this specific piston size.

The Cochlear system allows the determination of a threshold determined using the device called in situ threshold. The in situ threshold is psychophysically determined by controlling the level of the input voltage to the actuator and hence the vibrational output level of the actuator to the cochlea until the implanted patient detects and confirms an audible signal. This procedure is similar to pure tone audiometry and determines the threshold in device specific units. Although in situ thresholds are an important and valuable tool in clinical practice, allowing insight to e.g. long-term stability of the sound transmission, they depend on multiple factors and vary substantially between subjects. In our study, the frequency specific input voltage to the actuator ${u(f)}_{THR}^{i}$ at the psycho-physical threshold at frequency f was determined from the in situ measurements of each individual, using technical data (provided by Cochlear Ltd.). The frequency dependent electro-mechanical transfer function of the actuator is:

2 $${H(f)}_{EM}=\frac{d}{E}$$

where d is the displacement of the actuator and E is the electrical input voltage. From Eq. (2) and ${u(f)}_{THR}^{i}$ the displacement output that led to an audible signal in a patient (with index i) at a frequency f can be calculated.

3 $${d(f)}_{THR}^{i}={{H(f)}_{EM} u(f)}_{THR}^{i}$$

As the choice of the correct electro-mechanical transfer function (Eq. 2) is crucial, we investigated two alternative options: (A) using the (unloaded) electro-mechanical transfer function ${H(f)}_{EM}^{bench}$ as it is measured during manufacturing on the bench, and (B) using the electro-mechanical transfer function ${H(f)}_{EM}^{IOP}$ as it is measured intraoperatively by LDV using a known electrical input stimulus E.

#### Electro-mechanical transfer functions ${H(f)}_{EM}^{bench}$ from bench measurements

**Figure 2:**
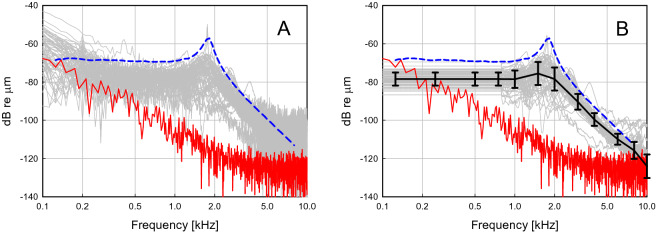
(**A**) Intraoperative measurement of the actuator displacement output (100 averages) when driven through a modified sound processor with pseudo-random white noise (12.5 Hz resolution 800 FFT lines) of − 46.5 dB V/line. The standard displacement output before surgery on the bench (dashed blue line) at the same input and individual displacement responses in patients (N = 66) measured intraoperatively (raw transfer function, grey). The red line depicts an example of a common noise floor level during an intraoperative measurement. (**B**) Same measurements after application of the smoothing procedure (smoothed transfer function, grey) as described in the section ‘Electro-mechanical transfer function from intraoperative measurements’ and averaged values with standard deviations (black).

Initially, in actuators used during the clinical trial an actuator specific electro-mechanical transfer function determined on the bench for each actuator was used. In devices implanted after the CE approval, a common electro-mechanical transfer function determined on the bench was applied (see blue line Fig. 2). All technical data were provided by Cochlear Ltd.

#### Electro-mechanical transfer functions ${H(f)}_{EM}^{IOP}$ from intraoperative measurements

**Figure 3:**
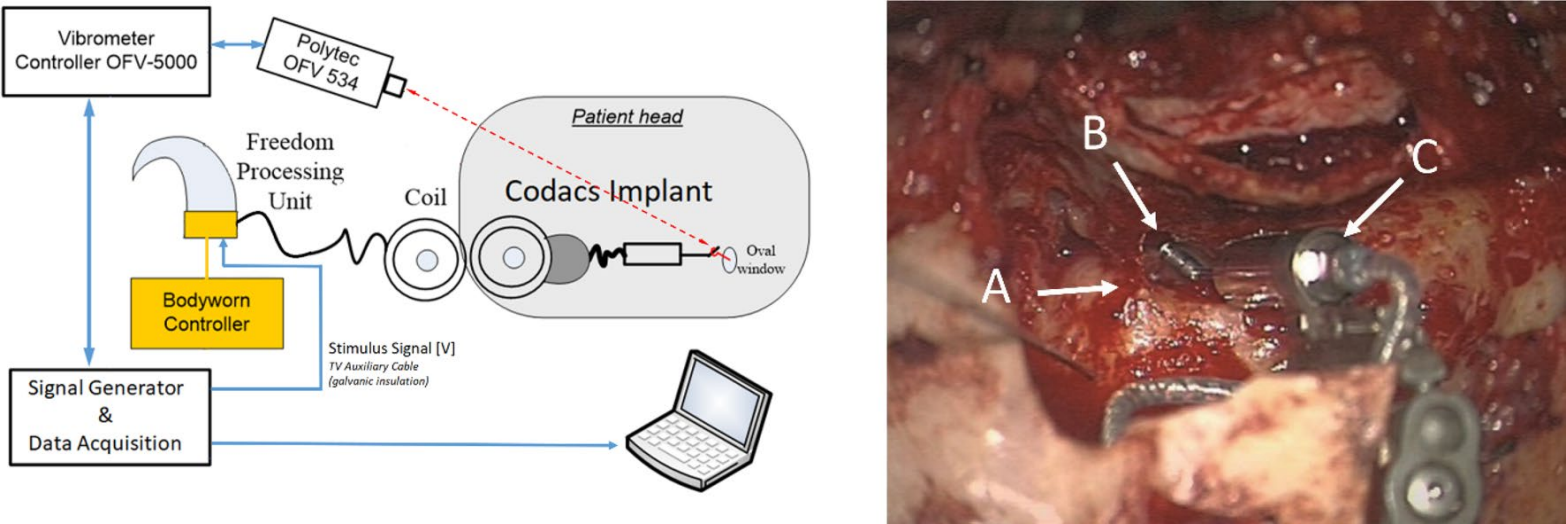
Left panel: Setup for the intraoperative measurement of the electro-mechanical transfer function. The white noise signal is generated on a computer controlled data acquisition system driving the input of a modified implant processor. The vibration response of the artificial incus of the actuator is measured by the laser Doppler vibrometer and recorded with the data acquisition system. Right panel: Surgical situation during intraoperative measurement: (A) piston prosthesis reaching into the cochlea through a stapedotomy, (B) artificial incus of the actuator, (C) Codacs actuator body.

During surgery (after implantation of the Codacs, but before closure of the surgical site), the integrity of the device was tested (Fig. 3). For this purpose, a custom-made sound processor was connected to the transmission coil and driven with an externally generated frozen white noise signal (12.5 Hz–12.8 kHz, 12.5 Hz FFT resolution, 100 averages) and the actuator output was measured by laser Doppler vibrometry (LDV) on the artificial incus of the implant.

In (A) it is assumed that the properties of the actuators measured during manufacturing on the bench are not changed during the implantation. This is usually not the case and is easily detectable if a comparison between the standard electro-mechanical transfer function and the transfer function measured intraoperatively is made (Fig. 2). However, the raw transfer functions measured intraoperatively are noisier than on the bench and suffer from an elevated noise floor. Thus, the following corrections were performed to obtain a filtered version of the intraoperative transfer functions.

1. The displacement output was approximately constant below the resonance at approx. 1.8 kHz. As the displacement output measured intraoperatively at low and high frequencies was mostly not sufficiently above the noise floor (< 12 dB, see Fig. 2A), the output level below 500 Hz was assumed to be constant. Here, the level was estimated from the average in the plateau range between 500 and 800 Hz, which was usually sufficiently above the noise floor. Values below 800 Hz were replaced by this estimate.
2. At mid-frequencies between 0.8 and 3 kHz, where usually a sufficient signal-to-noise-ratio is seen in intraoperative measurements, a 5-point moving average was applied to smooth the data.
3. At frequencies > 3 kHz, a 21-point moving average was applied to smooth the data.
4. Bench and intraoperative data were measured under slightly different conditions. While bench data were measured parallel to the actuator axis, intraoperative measurements were performed perpendicular to the ‘artificial incus’, resulting in a 25° angle to the axial direction of the actuator. Because the LDV measures only in the direction of the laser beam the displacement in axial direction was calculated by dividing the measured displacement by the cosine of this angle.

Data sets of 66 intraoperatively measured patients were available for the analysis performed here. Using the smoothed intraoperative electro-mechanical transfer functions, the difference to the standard transfer function on the bench was calculated.

Obviously, audiometric and in situ thresholds will depend on the individual inner ear (bone conduction) hearing threshold ${BC(f)}_{i}$. We assumed that the generation of the cochlear stimulus, i.e. the pressure difference across the basilar membrane, is similar for piston stimulation and bone-conducted sound. Consequently, ${BC(f)}_{i}$, that is expressed in dB HL can be used to correct the displacement at threshold for a sensorineural hearing loss component to obtain a hypothetical normal hearing test subject displacement:

4 $${d(f)}_{0 dBHL}^{i}={d(f)}_{THR}^{i}{-BC(f)}_{i}$$

Referencing all displacements to a hypothetical common threshold value allows for a statistical analysis of the cohort. Middle ear transfer functions (METFs) are usually determined with the sound pressure reference at the tympanic membrane^1^. The displacement at threshold from Eq. (4) is given in dBHL in sound field and requires the conversion from dBHL to dBSPL and from sound field to sound pressure level at the tympanic membrane. Hence, the displacement amplitude results at hearing threshold in Eq. (4) were converted to dB SPL at the tympanic membrane (${d(f)}_{0 dBHL}^{i}\to {d(f)}_{0 dBSPL}^{i}$) using the conversion tables from^6,7^ for the respective steps. Finally, the displacement for 94 dB SPL at the tympanic membrane was determined for comparison with an established middle ear transfer function^8^.

Additionally, for better comparison with the maximum output level of HAs (output sound pressure level at 90 dB SPL input signal; OSPL90^9^) the equivalent sound pressure in sound field [eq dB SPL_FF_] was calculated, from the same patient data. For this purpose, the frequency specific input voltage to the actuator ${u(f)}_{THR}^{i}$ at the in situ threshold of an individual (index i) was used. Assuming again the correspondence with the inner ear threshold ${BC}_{i}$ and linearity of the electro-mechanical transfer function of the actuator, the output level at 1V_rms_ input was calculated. For conversion from dBHL to dBSPL_FF_, the Reference Equivalent Threshold Sound Pressure Levels (RETSPLs) for sound-field testing from ANSI 3.6-2004 was used^6^.

### Ethics declaration

All patient data was acquired during routine measurements and the analysis was performed in retrospective from patient files and intraoperatively recorded data. Informed consent for anonymous use of data was obtained at the admission of patients. The data processing was done anonymous in accordance to the Regulation (EU) 2016/679 of 27 April 2016 on the protection of natural persons with regard to the processing of personal data, relevant guidelines and regulations and the internal regulations of the institution (MHH). According to German Data Protection and Professional Laws an ethics committee approval was not necessary for this study design as confirmed in a written statement by the local ethics committee.

## Results

To quantify the impact of the implantation and loading on actuator performance, the difference between actuator displacement on the bench and measured intraoperatively after implantation was determined. Measurement conditions for intraoperative data have usually less than optimal conditions, leading to low signal-to-noise-ratios (SNRs), which made an averaging/smoothing of data at high and low frequencies necessary (for details see methods). Intraoperative data were available from 66 patients. The differences between intraoperative data and bench data at audiometric frequencies are shown in Table 2.

**Table 2:** Displacement amplitude difference between standard actuator response measured unloaded on the bench to actuator displacement measured intraoperatively after implantation (N = 66).

Frequency (kHz)	0.125	0.25	0.5	0.75	1.0	1.5	2.0	3.0	4.0	6.0	8.0
Mean (dB)	− 9.3	− 9.2	− 8.6	− 8.5	− 8.8	− 10.7	− 13.6	− 5.7	− 5.9	− 4.4	− 1.9
Median (dB)	− 9.2	− 9.2	− 8.5	− 8.4	− 8.1	− 9.9	− 12.4	− 6.2	− 5.7	− 4.3	− 2.6
25th percentile (dB)	− 11.2	− 11.1	− 10.5	− 10.4	− 11.0	− 12.8	− 15.8	− 7.5	− 6.8	− 5.9	− 4.1
75th percentile (dB)	− 7.0	− 6.9	− 6.3	− 6.2	− 5.9	− 6.2	− 9.5	− 3.9	− 4.3	− 3.1	− 0.4
5th percentile (dB)	− 15.1	− 15.1	− 14.4	− 14.3	− 18.9	− 23.4	− 25.5	− 11.0	− 10.3	− 7.8	− 8.0
95th percentile (dB)	− 3.8	− 3.8	− 3.2	− 3.1	− 1.5	− 2.4	− 5.8	1.7	− 2.0	0.0	8.5

A decrease in output amplitude by the surgery is indicated by negative values while an increase results in a positive one.

Differences were not normally distributed above 1 kHz (Shapiro–Wilk) and median values were between 12.4 and 2.6 dB (p < 0.001 at all frequencies, Mann–Whitney Rank Sum Test). The maximum differences were found at 1.5–2 kHz. An upshift of the resonance frequency, that is at approximately 1.8 kHz in unloaded bench measurements, is a common observation after (surgical) manipulation. In intraoperative measurements the increase of the resonance is also used as a monitor for the integrity of the actuator. Resonances above approximately 2.2 kHz are an indicator that the transducer was damaged during implantation and needs to be replaced.

**Figure 4:**
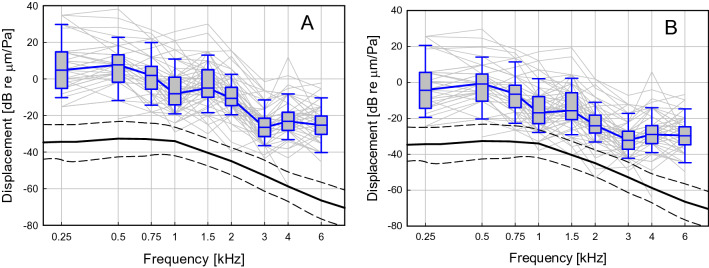
Estimated piston displacement of all patients (N = 77) that evokes a perception equivalent to a sound stimulation of 94 dBSPL at the tympanic membrane. Panel (**A**) shows the median piston displacement (blue) if an electro-mechanic transfer function of the actuator as measured unloaded on the bench is assumed. Panel (**B**) shows the same as before but for an electro-mechanic transfer function corrected for intraoperative conditions. In both panels piston displacements of individual patients as shown as thin grey lines. Box plots show median responses with the 25th/75th percentile range and whiskers depict the 5th/95th percentiles. The solid black line depicts the average stapes footplate displacement in normal ears at 94 dB SPL and the dashed black lines the 5th/95th percentile range^8^.

For the determination of the piston displacement with the Codacs necessary to evoke a perception equivalent to 94 dB SPL at the tympanic membrane, data sets of 77 ears were available. At most frequencies, the data were not normally distributed (Shapiro–Wilk), neither for the electro-mechanical transfer function determined on the bench ${H(f)}_{EM}^{bench}$, nor for the intraoperative determined ${H(f)}_{EM}^{IOP}$. For both cases, individual and resulting median displacements are shown in Fig. 4 and listed in Table 3.

**Table 3:** Piston displacement amplitude for 94 eq dBSPL at the tympanic membrane of all patients (N = 77).

Frequency (kHz)	0.25	0.5	0.75	1.0	1.5	2.0	3.0	4.0	6.0
N	39	60	53	69	73	72	69	66	59
**Reference bench**									
Mean (dB re μm)	7.4	6.2	1.2	− 5.2	− 2.6	− 10.1	− 25.5	− 21.4	− 23.8
Median (dB re μm)	4.8	7.7	1.9	− 8.1	− 5	− 10.6	− 26.5	− 23.2	− 25.3
25th percentile (dB re μm)	− 5.2	− 1.8	− 5.6	− 14.1	− 10	− 14.6	− 31.5	− 28.2	− 30.3
75th percentile (dB re μm)	14.8	13.2	6.9	0.9	5	− 4.6	− 21.5	− 18.2	− 20.3
10th percentile (dB re μm)	− 10.2	− 11.8	− 14.3	− 19.1	− 18.4	− 19.6	− 36.5	− 33.2	− 40.3
90th percentile (dB re μm)	29.8	22.7	19.9	10.9	13	2.5	− 11.5	− 8.2	− 10.3
**Reference IOP**									
Mean (dB re μm)	− 1.7	− 2.4	− 7.2	− 14.1	− 13.3	− 23.7	− 31.2	− 27.2	− 28.2
Median (dB re μm)	− 4.4	− 0.9	− 6.6	− 16.9	− 15.7	− 24.2	− 32.2	− 29.1	− 29.7
25th percentile (dB re μm)	− 14.4	− 10.4	− 14.1	− 22.9	− 20.7	− 28.2	− 37.2	− 34.1	− 34.7
75th percentile (dB re μm)	5.6	4.6	− 1.6	− 7.9	− 5.7	− 18.2	− 27.2	− 24.1	− 24.7
10th percentile (dB re μm)	− 19.4	− 20.4	− 22.8	− 27.9	− 29.1	− 33.2	− 42.2	− 39.1	− 44.7
90th percentile (dB re μm)	20.6	14.1	11.4	2.1	2.3	− 11.1	− 17.2	− 14.1	− 14.7

Piston displacement amplitudes necessary to evoke a percept equivalent to 94 dB SPL at the tympanic membrane were highly variable and differed significantly between individuals. The interquartile range was between 10 and 20 dB and the 10th/90th percentile was between 22 and 40 dB. Nevertheless, the general frequency characteristics of the median results were similar to the middle ear transfer function with a plateau at low frequencies (< 1 kHz) and a decrease at high frequencies. The displacement, necessary for a 94 dBSPL equivalent with a 0.4 mm piston was significantly higher (p < 0.001, Mann–Whitney Rank Sum Test) at all frequencies, independent of whether the electro-mechanical transfer function ${H(f)}_{EM}^{bench}$ or ${H(f)}_{EM}^{IOP}$ was used for calculation. If the preoperative bench measurement was used, the difference between the mean stapes footplate^1,8^ and piston displacement ranged from 28.8 to 42.6 dB, with an average of 35.9 dB across frequencies (0.25–6 kHz). When the intraoperative measured electro-mechanical transfer function was used for calculation the difference ranged from 20.0 to 38.4 dB (27.6 dB on average).

**Figure 5:**
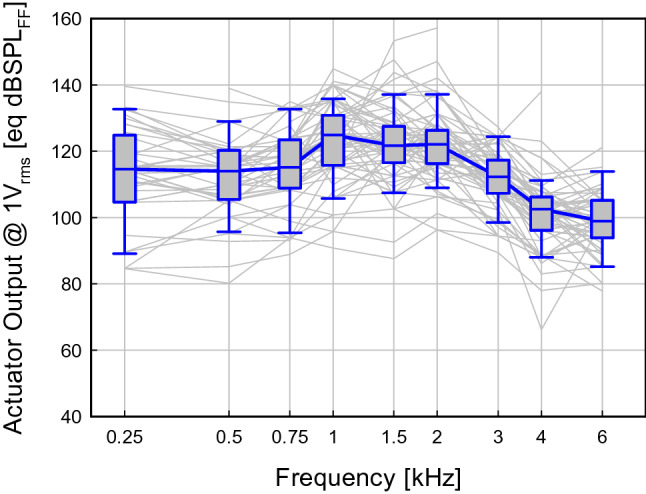
Estimated equivalent sound pressure in sound field [eq dB SPL_FF_] generated by the Codacs at an input of 1V_rms_ to the actuator. Box plots show median responses of all ears (N = 77) and thin grey lines individual results.

Additionally, the equivalent sound pressure output of the actuator in conjunction with a 0.4 mm piston of all ears was calculated from the same data. The result at a hypothetical input voltage of 1V_rms_ is shown in Fig. 5. The median output was between 110 and 130 eq dBSPL_FF_ up to 3 kHz and decreased to approximately 100 eq dBSPL_FF_ above. Individual results showed a large variability.

**Figure 6:**
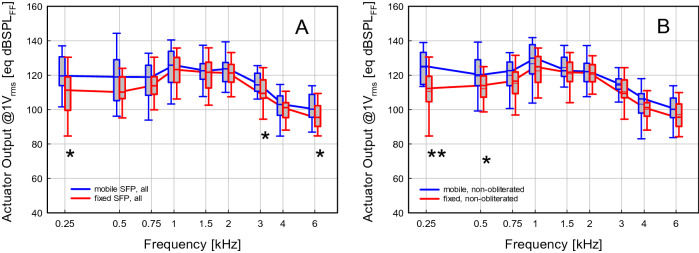
Estimated equivalent sound pressure in sound field [eq dB SPL_FF_] generated by the Codacs at an input of 1V_rms_ to the actuator. (**A**) Median responses in mobile (blue, N = 30) and fixed (red, N = 47) stapes footplates of all ears (N = 77). (**B**) Median responses in mobile (blue, N = 21) and fixed (red, N = 43) stapes footplates of non-obliterated ears (N = 64). *p < 0.05, **p = 0.01; Mann–Whitney Rank Sum Test.

As the condition of the middle ear and stapes footplate (SFP) of implanted ears differed, a refined analysis was performed. When ears with fixed footplates (N = 47) were compared to ones with mobile footplates (N = 30) (Fig. 6A), statistically significant differences were found at 0.25 (11.0 dB), 3 (5.2 dB) and 6 (6.1 dB) kHz (p < 0.05, Mann–Whitney Rank Sum Test). Comparison of obliterated (N = 13) to non-obliterated (N = 64) ears revealed no indication of significant differences. In the analysis of subgroups (1) fixed/non-obliterated, (2) mobile/non-obliterated, (3) fixed/obliterated, (4) mobile/obliterated statistically significant differences (Mann–Whitney Rank Sum Test) were found in the comparison of mobile vs fixed stapes footplates in non-obliterated ears (Fig. 6B). Ears with mobile SFP yielded a significant higher output amplitude at 0.25 kHz (14.1 dB; p = 0.01) and 0.5 kHz (7.4 dB; p < 0.05). The distribution in these subgroups can be assumed normally distributed (Shapiro–Wilk) and a post hoc power estimate (t-test; α = 0.05) indicated a power of 0.868 for both frequencies. In all other pair-wise comparison between subgroups (1)–(4) no significant effects were found.

## Discussion

The primary goal of our study was to determine the difference in coupling efficiency at the biological interface to the inner ear when the stapes footplate is replaced with an artificial piston of smaller active area driven by an actuator. Using clinical audiological data from a large cohort of patients, the displacement amplitude to evoke the same loudness percept was calculated for speech relevant frequencies (0.25–6.0 kHz). The actuator performance is subject to loading and changes related to the surgery. Hence, two different scenarios for the determination of the electro-mechanical transfer function of the actuator were assumed: (1) the unloaded electro-mechanic transfer function as measured during manufacturing on the bench and (2) the electro-mechanic transfer function measured intraoperatively at the end of the surgery. The two cases represent (1) the best case scenario and (2) the worst case scenario when the output is decreased by the mechanical load and the manipulation during surgery. Our results (Fig. 4, Table 3) indicate that the displacement magnitude follows the general characteristics of the middle ear transfer function (METF) of a healthy normal middle ear in response to a 94 dB SPL stimulus at the tympanic membrane, but with significant reduced stimulation efficiency. This means that the actuator has to generate a higher displacement output of the piston than the stapes footplate of the middle ear to produce the same loudness percept. Compared to the displacement amplitude necessary to evoke a percept equivalent to 94 dB SPL at the tympanic membrane the average transfer function (0.25–6 kHz) of the piston was found 35.9 dB higher when the electro-mechanical transfer function of the actuator on the bench (1) was assumed. When the intraoperatively measured electro-mechanical transfer function (2) was used the average piston needed only 27.6 dB higher displacement amplitudes at average which is close to the value expected from the ratio of the piston to the stapes footplate area.

Although the general frequency dependence was similar, mean differences and individual piston transfer functions varied substantially. At 1 kHz and 3 kHz differences were smaller, possibly due to an shift in actuator resonance (approximately 1.8 kHz in the unloaded, non-implanted state, see Fig. 2). At high frequencies the difference was larger, which might be attributed to a mismatch in slope between the actuator that has a roll-off above 2 kHz of − 12 dB/octave^10^ and the METF that has a slightly steeper roll-off (< − 12 dB/octave^8^). However, our main finding here was that the average efficiency of the 0.4 mm piston can be expected between − 27.6 and − 35.9 dB which is less than the normal sound transduction through the stapes footplate. This is also in good accordance with the geometrical assumption comparing the volume displacing areas of the piston with the stapes footplate. The surface areas of the piston (0.126 mm^2^) and the stapes footplate of 3.2 mm^2^ reported in the literature^11,12^ yield a ratio of 28.1 dB that falls well into the range found here from clinical data.

The actuator has a relatively large force reserve available and the output remains mainly unaffected by the load. In experiments, embedding the actuator in a stiff material to simulate tissue growth no major effect on output amplitude and resonance frequency was found^13^. This and the observation that the resonance frequency is not shifted at all in a large number of intraoperative measurement (Fig. 2) indicates that shifts in resonance frequency are probably not caused by the load but by the manual handling during the implantation procedure. This is in accordance with our own experience of changes in actuator characteristics in experiments. In turn, this leads to the conclusion that the lower value of approximately − 28 dB, derived from the intraoperative measurements is a more realistic estimate of the piston efficiency compared to the stapes footplate of a normal middle ear. Nevertheless, the upper limit of approximately − 36 dB, based on the transfer function measured on the bench is a valuable indicator for the reserve that has to be considered for the specifications of new actuator designs. In active, actuator based middle ear reconstructions with implants the reduction in stimulation efficiency can be easily compensated by the gain. Even more importantly, the usually pronounced variability in coupling efficiency can be postoperatively adjusted to the individual needs of the patient without surgical intervention.

It needs to be emphasized that these results are valid for the biological interface to the cochlea for direct acoustic stimulation with an actuator and do not apply to surgical reconstruction such as stapesplasty with the same prosthesis. This is in contrast to surgical reconstruction of the middle ear with the same or another piston diameter. By replacing the ossicles with a total ossicular replacement prosthesis (TORP) not only the input efficiency is changed, but the entire mechanical middle ear transformation process is altered, including the load at the tympanic membrane. In the acoustic/mechanical analog model by Rosowski and Merchant^14^ the decrease in transmission from the TM to the cochlea, i.e. the resulting air-bone-gap (ABG) is calculated. While the model prediction for a piston diameter of 1.0 mm leads to a minor ABG, it leads for a 0.4 mm diameter to an ABG of 16–18 dB in the frequency range between 0.1 and 10 kHz. Although this model uses the same geometrical piston/SFP ratio at the oval window, the predicted ABG is much smaller because positive and negative effects partially cancel out. This is not the case if an actuator is used as input element. In surgical reconstructions with a TORP, such as stapedotomy, a common success criterion is a postoperative ABG ≤ 10 dB^15^, but clinical success rates vary in a broad range^16^. Although a vast amount of clinical data is available, results are highly heterogeneous and publications that find a piston size effect on AGB^17,18^ are contradicted by other publications that find no significant influence of piston size. Even experiments with a similar actuator did not find a size effect comparing 0.5 and 0.8 mm pistons^5^. Our findings strongly support the assumption of a geometrical dependency on piston size at the biological interface to the cochlea. These results imply major practical consequences for the understanding of existing devices and the design of new actuators that stimulate the inner ear directly.

In addition to the estimation of the efficiency at the cochlea interface, we used our approach to determine the equivalent output level from clinical data. In contrast to experiments, this allows to determine the output level in a clinically relevant postoperative situation when the piston and the actuator are embedded in tissue and the cochlea opening is sealed by mucosa. Under these realistic conditions the median output level of the device was approximately 110 ± 10 eq dB SPL_FF_ up to 3 kHz and decreased to approximately 100 eq dB SPL_FF_ at higher frequencies (Fig. 5). This is in good accordance with earlier findings that estimated the output of the device as > 112 dB SPL with another experimental method in a small number of patients^4^. A detailed analysis indicated that the achieved output significantly increased to > 120 eq dB SPL_FF_ at low frequencies by a mobile footplate (Fig. 6). In contrast, obliteration of the middle ear had no significant effect on output level indicating that embedding in fatty tissue has no impact on the actuator or the piston.

The advantage of the method employed here is that it requires only data available from clinical routine, i.e. BC and in situ thresholds. It also works with threshold data and allows determination of the maximum power output (MPO) even if it is above the UCL of the patient. Other available methods, based on saturation level for bone conduction devices (BCDs)^19^ and other active middle ear implants (AMEIs) require additional complex non-routine measurements and setups^3^. Additionally, in these methods the saturation level close to the MPO has to be measured and yields only estimates if the MPO is above the UCL or levels that are too loud to be applied without the risk of noise damages.

In summary, our data provides first clinical/experimental evidence that the efficiency of an actuator driven piston at the biological interface to the cochlea can be estimated using the ratio between the surface area of the piston to the stapes footplate. Under realistic in vivo conditions with 1 V_rms_ input individual maximum power output was highly variable. At average the achieved output level of the Codacs actuator was ~ 110 ± 10 eq dB SPL_FF_ below 3 kHz, decreasing to ~ 100 eq dB SPL_FF_ at 10 kHz. The achieved output has significantly increased at low frequencies when the footplate was mobile, whereas obliteration of the middle ear with fatty tissue had no detectable effect on output level.

## Data Availability

The anonymized data used to support the findings of this study are available from the corresponding author upon request.
